# Delivery Room ST Segment Analysis to Predict Short Term Outcomes in Near-Term and Term Newborns

**DOI:** 10.3390/children9010054

**Published:** 2022-01-03

**Authors:** Jørgen Linde, Anne Lee Solevåg, Joar Eilevstjønn, Ladislaus Blacy, Hussein Kidanto, Hege Ersdal, Claus Klingenberg

**Affiliations:** 1 Department of Obstetrics and Gynecology, Stavanger University Hospital, 4068 Stavanger, Norway; 2 Department of Pediatric and Adolescent Medicine, Oslo University Hospital, 0424 Oslo, Norway; a.l.solevag@medisin.uio.no; 3 Laerdal Medical and Laerdal Global Health, 4002 Stavanger, Norway; Joar.Eilevstjonn@laerdal.com; 4 Haydom Lutheran Hospital, Private Bag Mbulu, Haydom P.O. Box 9000, Tanzania; lblacy@haydom.co.tz; 5 Medical College, Agakhan University, Dar es Salaam P.O. Box 38129, Tanzania; hussein.kidanto@aku.edu; 6 Faculty of Health Sciences, University of Stavanger, P.O. Box 8600, 4036 Stavanger, Norway; hege.ersdal@safer.net; 7 Critical Care and Anaesthesiology Research Group, Stavanger University Hospital, 4068 Stavanger, Norway; 8 Pediatric Research Group, Faculty of Health Sciences, UiT—The Arctic University of Norway, 9019 Tromsø, Norway; Claus.Klingenberg@unn.no; 9 Department of Pediatrics and Adolescent Medicine, University Hospital North Norway, 9019 Tromsø, Norway

**Keywords:** Electrocardiography, bag-mask ventilation, perinatal asphyxia, ST-elevation

## Abstract

Background: ST-segment changes to the fetal electrocardiogram (ECG) may indicate fetal acidosis. No large-scale characterization of ECG morphology immediately after birth has been performed, but ECG is used for heart rate (HR) assessment. We aimed to investigate ECG morphology immediately after birth in asphyxiated infants, using one-lead dry-electrode ECG developed for HR measurement. Methods: Observational study in Tanzania, between 2013–2018. Near-term and term infants that received bag-mask ventilation (BMV), and healthy controls, were monitored with one-lead dry-electrode ECG with a non-diagnostic bandwidth. ECGs were classified as normal, with ST-elevations or other ST-segment abnormalities including a biphasic ST-segment. We analyzed ECG morphology in relation to perinatal variables or short-term outcomes. Results: A total of 494 resuscitated and 25 healthy infants were included. ST-elevations were commonly seen both in healthy infants (7/25; 28%) and resuscitated (320/494; 65%) infants. The apparent ST-elevations were not associated with perinatal variables or short-term outcomes. Among the 32 (6.4%) resuscitated infants with “other ST-segment abnormalities”, duration of BMV was longer, 1-min Apgar score lower and normal outcomes less frequent than in the resuscitated infants with normal ECG or ST-elevations. Conclusions: ST-segment elevation was commonly seen and not associated with negative outcomes when using one-lead dry-electrode ECG. Other ST-segment abnormalities were associated with prolonged BMV and worse outcome. ECG with appropriate bandwidth and automated analysis may potentially in the future aid in the identification of severely asphyxiated infants.

## 1. Introduction

Heart rate (HR) assessment is essential when evaluating a compromised newborn infant immediately after birth. Most depressed infants with a HR < 100 beats per minute respond with an increase in HR to basic newborn resuscitation measures including drying, stimulation and mask ventilation [1]. However, some infants experience a more severe hypoxic-ischemic insult leading to impaired oxygen supply to the myocardium, neonatal encephalopathy and/or death [2,3].

In recent years, ST-analysis (STAN) of the fetal electrocardiogram (ECG) has been used in combination with cardiotocography (CTG) for surveillance of fetal wellbeing. Fetal metabolic acidosis is suspected when an abnormal CTG is accompanied by changes in the ST-segment of the fetal ECG [4]. A Cochrane review of CTG with STAN reported that enhanced monitoring with both techniques leads to reduced need for admission to the neonatal unit as well as less instrumental vaginal deliveries [5].

Immediately after birth, no standardized delivery room evaluation of myocardial compromise has been possible [6]. Indeed, the current way to analyze ST-segment changes with a traditional diagnostic bandwidth (0.05–150 Hz) 12-lead ECG is not suitable for use in the delivery room. After admission to a neonatal unit, cardiac enzymes including troponins are used for diagnostic and prognostic work-up [7,8]. Research to investigate other methods to assess myocardial involvement has been hampered by their resource intensiveness (e.g., echocardiography) or lack of feasibility (e.g., a need for mother-infant separation). ECG is now widely used as a reliable method for neonatal HR assessment in the delivery room [9,10]. Recently, easily applicable dry-electrode ECG designed for HR measurement has been introduced [11,12,13]. However, a potential limitation of ECG monitors for HR measurement is a more limited bandwidth than traditional 12-lead diagnostic ECG and STAN [14], which may lead to falsely elevated ST-segments [15].

We recorded one-lead dry-electrode ECG in a large cohort of infants resuscitated with bag-mask ventilation (BMV), and a healthy control group immediately after birth. We aimed to describe ECG morphology during the first minutes of life, and analyze the association between ECG morphology and short-term outcomes using this easily applicable equipment.

## 2. Materials and Methods

This observational study is part of Safer Births, a research project on labor surveillance and newborn resuscitation in low-income settings [16].

### 2.1. Study Design and Participants

**Figure 1:**
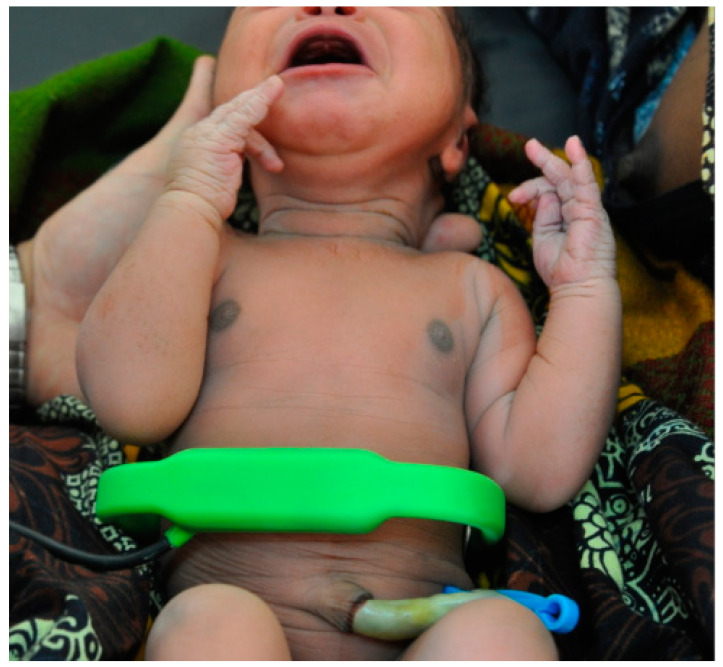
The dry-electrode ECG sensor used in this study.

We used data collected between 01.07.13 and 30.06.18 at Haydom Lutheran Hospital, a rural Tanzanian referral hospital with 3.600–4.600 annual deliveries [17]. Newborn resuscitation was mainly the responsibility of midwives. All newborn infants were dried thoroughly after birth, and non-breathing neonates were stimulated before the umbilical cord was clamped and cut. Neonates not responding to stimulation were transferred to the resuscitation table, and a dry-electrode ECG sensor were placed around the newborn’s trunk (Figure 1). The local procedure for newborn resuscitation followed the Helping Babies Breathe algorithm, emphasizing stimulation and early initiation of BMV, and did not include chest compressions or medication [18]. After stabilization, the midwives decided, based on the clinical condition, whether to keep the newborn with the mother or transferring the infant to a neonatal ward offering basic care, including antibiotics, phototherapy, and intravenous fluids, but no respiratory support, except for supplemental oxygen by nasal cannula [19].

**Figure 2:**
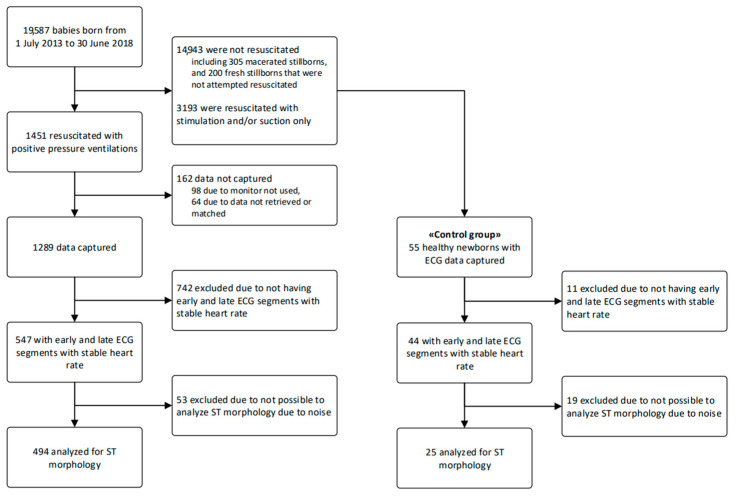
Flow diagram of inclusions.

For this study, term and near-term (from 34 weeks gestation) infants that received BMV and had concomitantly ECG recordings were eligible. See Figure 2 for the flow of study participants. We also included a control group (n = 44) of healthy infants, without any need for resuscitation after birth, but who had dry-electrode ECG placed for evaluation of HR.

### 2.2. Equipment and Data Collection

Newborn resuscitation monitors (Laerdal Global Health, Stavanger, Norway) with dry-electrode ECG were installed in every delivery room and in the operation theatre. The monitors started data recording automatically when used. The ECG bandwidth of the monitor was 1–150 Hz, and it was primarily designed to provide HR feedback. The ECG sensor was typically applied to the torso of the newborn to record ECG, roughly equivalent to lead I of a standard ECG. Trained non-medical research assistants observed all deliveries documenting perinatal information and time intervals [17]. Baseline perinatal variables included fetal HR assessment, obstructed labor on World Health Organization (WHO) partogram, delivery method, gender and birth weight.

### 2.3. Processing and Interpretation of the ECG

ECG signal data from included infants were extracted, and appropriate processing using Matlab R2020a (MathWorks Inc., Natick, MA, USA) was performed. The electronic ECG files were processed using a 50 Hz notch filter and zero-phase forward and reverse filtering to avoid phase distortion.

**Figure 3:**
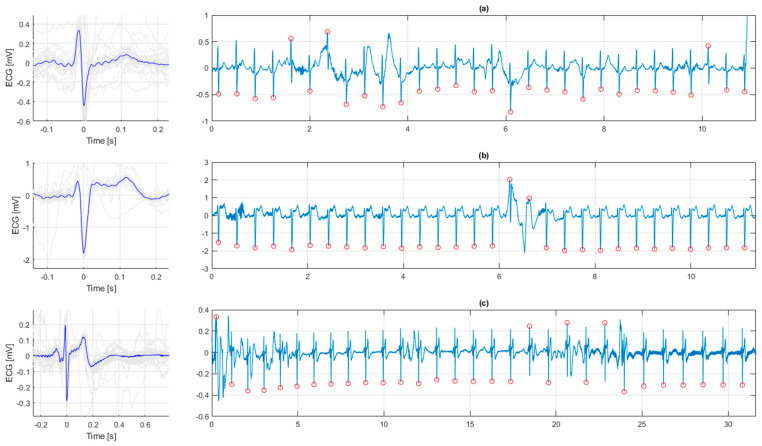
Examples of ECG morphologies. (**a**) Normal ECG. (**b**) ST elevation. (**c**) A biphasic ST-segment. Left in Figure 3a–c are the medians of all complexes aiding the visual analysis.

For each individual infant, we searched for and analyzed ECG for two periods (“early” and “late”), during which intra-period variation in HR was < 20 bpm. The early period was defined as the first 30 subsequent QRS-complexes fulfilling the intra-period variation requirement and recorded within 3 min after birth, and the late period as the final 30 subsequent QRS-complexes during HR assessment. ECG-complexes were aligned and overlaid to find the median QRS-complex to improve the signal-to-noise-ratio and construct a representative complex, shown in Figure 3.

Morphology of the ECG was interpreted by four researchers (JL, ALS, JE, CK) who were blinded to both the HR during the ECG recording and to the individual infant outcome. The early and late ECG episodes were independently classified into three categories; (i) normal, (ii) ST-elevation, or (iii) “other ST-segment abnormalities”, including ST-depression and biphasic ST-segment. Examples are shown in Figure 3. Disagreements in interpretations were solved by consensus.

### 2.4. Outcome Variables

The primary outcome was ECG morphology in relation to three types of short-term outcomes; death (fresh stillbirth or early neonatal death, i.e., within one week), admission to the neonatal unit and alive at one week of age, or “normal outcome” defined as not in need for admission to the neonatal unit. Secondary outcomes include the following “perinatal variables”; mode of delivery, obstructed labor, Apgar scores, HR data immediately after birth and duration of BMV.

### 2.5. Statistical Analyses

We used SPSS (IBM SPSS Statistics for Mac, version 26.0; IBM Corp., Armonk, NY, USA) and Matlab R2021a (MathWorks Inc., Natick, MA, USA) for statistical analyses. Continuous variables are presented as means with standard deviation (SD) or medians with 25 and 75 percentiles (p25, p75), as appropriate. Categorical variables are presented as numbers (%). Differences between infants with ECG in the different morphological ECG-categories were analyzed with Kruskal–Wallis (continuous variables) or Chi-squared test (categorical variables). A *p* < 0.05 was considered statistically significant.

### 2.6. Ethical Considerations

Ethical approval was granted by the National Institute for Medical Research in Tanzania (Ref. NIMR/HQ/R.8a/Vol.IX/1434) and the Regional Committee for Medical and Health Research Ethics in Western Norway (Ref.2013/110). All mothers were informed about the ongoing research. Depending on whether the infant was included in a randomized controlled trial, the mothers gave deferred oral informed consent.

## 3. Results

During the five-year study period, 19,587 neonates were born, of which 1451 were resuscitated with BMV. The flow of study participants is shown in Figure 2.

### 3.1. ECG in Healthy Newborn Infants

The ECG sensor was mounted on the infants directly after birth to provide normative HR-data from the time of birth and before and after cord clamping [11]. The ECG recordings of 44 healthy infants were often subject to movement artefacts due to handling and vigorous infants. Of 44 recordings, 19 (43%) were of insufficient quality for interpretations to be made. In the 25 infants with acceptable quality ECGs, 18 (72%) had normal ECG and 7 (28%) had ECG ST-segment elevation. No healthy newborn had ECG classified as “other ST-segment abnormality”.

### 3.2. ECG in Resuscitated Newborn Infants

We included ECGs from 547 near-term and term resuscitated infants (Figure 2). After a thorough evaluation of the ECGs from these infants, we could not interpret ST-characteristics in 53 (10%), and we included 494 ECGs in the final analysis.

The early episodes of ECG were analyzed as soon as the ECG was obtained with acceptable quality and low intra-period HR variation, at a median of 117 (92, 143) seconds after birth. The late episodes of ECG assessment were analyzed at the very end of the resuscitation and stabilization episodes, at a median of 359 (238, 578) seconds after birth.

In 125 (25%) infants ECGs were normal both in the early and late period. In 311 (63%) infants the ECG appeared to have ST-segment abnormalities (including ST-elevations and/or “other ST-segment abnormalities”) both in the early and late period. In 32 infants the ST-segment changed from abnormal in the early to normal in the late period. In 16 infants a normal ECG changed to abnormal from the early to the late period. In 10 infants, the ECG was only possible to interpret in the early period (9 had ST-segment changes, and 1 was normal). In 4 infants the ECG was only possible to interpret in the late period (3 had ST-segment changes and 1 was normal). We did not observe any infant with ventricular fibrillation (VF) at onset of or during resuscitation.

### 3.3. Association between ECG Morphology and Perinatal Outcomes

Perinatal and neonatal characteristics in relation to ECG morphological category, based on the early episodes, are presented in Figure 1. A biphasic ST-segment comprised the main finding in the “other ST-segment abnormality” category, as exemplified in Figure 3c. There was no difference in rates of obstructed labor between infants in the three ECG groups, but infants delivered by caesarean (C-) section had a higher rate of “other ST- abnormalities” in their ECG (Table 1). In the infants with “other ST-segment abnormalities”, the duration of BMV was significantly longer, HRs were lower, 1-min Apgar score was lower and rates of normal outcome were lower than in infants with normal ECG or with ST-elevations (Table 1). Table 2 shows characteristics of infants in the three major clinical outcome categories. Infants who died were more frequently delivered by C-section, had higher rates of bradycardia and were resuscitated longer with BMV. The rate of “other ST abnormalities” was also higher in infants who died. In contrast, we found no association between ST-elevations and clinical outcomes.

## 4. Discussion

In this observational study, we present data on morphological analysis of one-lead dry-electrode ECG with a limited bandwidth from the first minutes of life. ST-segment elevations were observed in 28% of a control group of healthy infants, and in more than 60% of infants resuscitated after birth. In the latter group we found no associations between ST-elevations and perinatal variables or short-term outcomes. However, infants with “other ST-segment abnormalities”, in particular biphasic pattern, were ventilated longer after birth and had lower rates of normal outcome.

The equipment used for capturing ECG after birth had a limited 1–150 Hz bandwidth and was primarily designed for HR measurement and not ST-segment analysis. The 1–150 Hz bandwidth constitutes a high pass filter that may lead to a drift of the ST-segment that is falsely interpreted as ST-segment elevation, but in the absence of myocardial ischemia [15]. The device’s intended purpose is HR registration where the 1 Hz lower corner frequency is beneficial for reducing movement artefacts commonly observed during neonatal resuscitation. ST-elevations were indeed more commonly observed in resuscitated infants versus healthy controls, but in the absence of an association with clinical outcomes we speculate that the frequently occurring ST-segment elevations in our material at least partly may represent “false” ST-elevations.

In contrast, a biphasic ST-segment or other ST-segment abnormalities were associated with indicators of perinatal asphyxia including a lower HR after birth, need for a longer period of BMV, and lower rates of normal outcome. These ECG changes are potentially not subjected to the same possible error in the equipment used. Barberi et al. [20] reported that the most severe ECG findings [21], i.e., a flat or inverted T-wave, ST-depression or -elevation and abnormal Q-waves, were observed only in the group of severely asphyxiated infants. However, in their study, the ECG was taken at 24–36 h of age. Our analysis approach and results are more in line with the fetal STAN-classification, where biphasic ST-segment changes signify more severe asphyxia [22]. Notably, STAN utilizes a 0.05 to 100 Hz bandwidth [14]. In newborn piglet cardiac arrest studies, high frequencies of VF have been observed [23]. We did not observe VF in our study, but the number of neonates with severe asphyxia was limited. Moreover, swine are known to be more arrhythmogenic than humans, which may also explain why we did not observe cases with VFs [24].

The strength of our study is the large sample size of infants that were resuscitated with BMV and had ECG recordings of adequate quality for visual analysis. No other clinical study has evaluated ECG morphology immediately after birth in a large number of infants in need of BMV. Studies that evaluated ECG morphology the first or second day after perinatal asphyxia are mainly with small numbers and several decades old [20,21]. The major limitation is that we used ECGs from a monitor with a non-diagnostic bandwidth that may have resulted in “false” ST-elevations. Vigorous stimulation procedures were reasons for poor ECG quality, and visual analysis and classification based on ECG patterns are challenging. We decided on a crude three-level ECG classification that allowed a variety of ST-elevations to be classified in the same group. A standard ECG with neonatal electrodes was not available in this setting Moreover, we did not differentiate between subtle and more marked changes to the ST-segment and we did not include specific classification of the Q-waves. However, such further differentiation was not feasible given the signal noise often present in the ECGs in our setting with handling and resuscitation immediately after birth. The rural, low-income study site setting did not allow us to perform biochemical studies or echocardiography assessment of myocardial injury. Finally, the control group of healthy infants was small. Despite these limitations, our study generates hypotheses to be further investigated.

## 5. Conclusions

ST-segment elevations may not be a good proxy for severity of asphyxia when recorded with a non-diagnostic bandwidth ECG device intended for HR measurement. In contrast, biphasic ST-segment abnormalities are associated with bradycardia after birth and a need for prolonged BMV and might indicate significant asphyxia when this equipment is being used. Currently, automated systems for ECG classification are established for both adult [23] and fetal [22] ECG. Automated evaluation of the neonatal ECG, with appropriate bandwidth and automated analysis should be validated in future studies in order to evaluate whether this potentially can aid in the identification of severely asphyxiated infants.

## Figures and Tables

**Table 1:** Baseline characteristics and outcomes of resuscitated infants (n = 494) with three different ECG morphological findings.

	Normal ECG (n = 142)	ST Elevation (n = 320)	Other ST-Segment Abnormalities (n = 32)	*p*-Value ^1^
ANTENATAL CHARACTERISTICS				
Obstructed labor *	19 (13)	34 (11)	3 (9)	0.65
Vaginal head delivery	73 (51)	186 (58)	11 (34)	0.02
Caesarean section	63 (44)	119 (37)	20 (63)	0.01
Vaginal breech delivery	4 (3)	11 (3)	1 (3)	0.94
Vacuum	2 (1)	4 (1)	0 (0)	0.80
NEONATAL CHARACTERISTICS				
Gender (male)	70 (49)	207 (65)	14 (44)	0.002
Birth weight (g)	3155 (2750, 3500)	3290 (2990, 3600)	3110 (2943, 3415)	0.06
Apgar 1 min	7 (5, 7)	7 (5, 7)	5 (3, 7)	0.01
Apgar 5 min	10 (8, 10)	10 (8, 10)	9 (7, 10)	0.09
First heart rate [bpm]	128 (76, 165)	129 (74, 163)	66 (53, 134)	<0.001
Time from birth to ST analysis [s]	120 (95, 151)	116 (91, 142)	107 (89, 135)	0.21
Heart rate during ST analysis [bpm]	160 (139, 173)	157 (137, 172)	96 (56, 162)	<0.001
Resuscitation and outcomes				
BMV duration (s)	126 (68, 269)	143 (63, 281)	305 (117, 857)	0.002
Normal outcome	83 (58)	187 (58)	11 (34)	0.03
Admitted neonatal unit	44 (31)	97 (30)	13 (41)	0.49
Death-total	15 (11)	36 (11)	8 (25)	0.06
Classified as fresh stillbirth	1 (1)	4 (1)	1 (3)	0.53
Death within 24 h	9 (6)	21 (7)	5 (16)	0.15
Death from 1–7 days	5 (4)	11 (3)	2 (6)	0.72

^1^*p* values analyzed with Kruskal–Wallis or Chi-squared test. BMV—bag mask ventilation; bpm—beats per minute. Fresh stillbirths with ECG are misclassified by the observer. * Obstructed labor on the World Health Organization partogram.

**Table 2:** Characteristics of 494 infants with three outcomes.

Feature	Normal (n = 281)	Admitted (n = 154)	Death(n = 59)	*p*-Value ^1^
FHR < 120 bpm or > 160 bpm	26 (9)	28 (18)	10 (17)	0.02
Caesarean section	96 (34)	71 (46)	35 (59)	<0.001
ST elevation	187 (67)	97 (63)	36 (61)	0.62
Other ST abnormalities	11 (4)	13 (8)	8 (14)	0.01
First HR < 60 bpm	24 (9)	25 (16)	20 (34)	<0.001
First HR 60–100 bpm	49 (17)	57 (37)	25 (42)	<0.001
First HR ≥ 100 bpm	208 (74)	72 (47)	14 (24)	<0.001
Apgar 1 min	7 (7, 8)	6 (4, 7)	4 (2, 6)	0.01
Apgar 5 min	10 (10, 10)	8 (6, 10)	6 (3, 10)	0.09
Duration of BMV [s]	95 (53, 172)	234 (96, 425)	479 (211, 1297)	0.002

^1^*p* values analyzed with Kruskal–Wallis or Chi-squared test. BMV—bag mask ventilation; bpm—beats per minute; FHR—fetal heart rate; HR—heart rate.

## Data Availability

Deidentified individual participant data will be made available to researchers whose methodologically sound proposal has been approved by the Scientific Steering Committee for Safer Births Study Group. Proposals may be submitted up to 36 months following article publication to hege.ersdal@safer.net.
